# Combined effects of dietary *Laminaria digitata* with alginate lyase on plasma metabolites and hepatic lipid, pigment and mineral composition of broilers

**DOI:** 10.1186/s12917-022-03250-3

**Published:** 2022-04-27

**Authors:** Mónica Mendes Costa, Paula Alexandra Lopes, José Miguel Pestana Assunção, Cristina Maria Riscado Pereira Mateus Alfaia, Diogo Francisco Maurício Coelho, Miguel Pedro Mourato, Rui Manuel Amaro Pinto, Maria Madalena Lordelo, José António Mestre Prates

**Affiliations:** 1 CIISA - Centro de Investigação Interdisciplinar em Sanidade Animal, Faculdade de Medicina Veterinária, Avenida da Universidade Técnica, Pólo Universitário do Alto da Ajuda, Universidade de Lisboa, 1300-477 Lisbon, Portugal; 2 Laboratório Associado para Ciência Animal e Veterinária (AL4AnimalS), Lisbon, Portugal; 3 LEAF - Linking Landscape, Environment, Agriculture and Food, Instituto Superior de Agronomia, Universidade de Lisboa, Tapada da Ajuda, 1349-017 Lisbon, Portugal; 4 iMed.UL, Faculdade de Farmácia, Universidade de Lisboa, Avenida Professor Gama Pinto, 1649-003 Lisbon, Portugal; 5 JCS, Laboratório de Análises Clínicas Dr. Joaquim Chaves, Avenida General Norton de Matos, 1495-148 Miraflores, Algés, Portugal

**Keywords:** *Laminaria digitata*, Carbohydrate-active enZymes, Plasma biochemical markers, Hepatic lipid composition, Broiler

## Abstract

**Background:**

The *Laminaria digitata* is an abundant macroalga and a sustainable feedstock for poultry nutrition. *L. digitata* is a good source of essential amino acids, carbohydrates and vitamins, including A, D, E, and K, as well as triacylglycerols and minerals, in particular iron and calcium. However, the few studies available in the literature with broilers document the application of this macroalga as a dietary supplement rather than a feed ingredient. No study has addressed up until now the effects of a high-level incorporation (> 2% in the diet) of *L. digitata* on plasma biochemical markers and hepatic lipid composition, as well as minerals and pigments profile in the liver of broilers. Our experimental design included one hundred and twenty Ross 308 male birds contained in 40 wired-floor cages and distributed to the following diets at 22 days of age (*n* = 10) for 15 days: 1) a corn-soybean basal diet (Control); 2) the basal diet plus 15% of *L. digitata* (LA); 3) the basal diet plus 15% of *L. digitata* with 0.005% of Rovabio® Excel AP (LAR); and 4) the basal diet plus 15% of *L. digitata* with 0.01% of the recombinant CAZyme, alginate lyase (LAE).

**Results:**

*L. digitata* compromised birds’ growth performance by causing a reduction in final body weight. It was found an increase in hepatic *n*-3 and *n*-6 fatty acids, in particular C18:2*n*-6, C18:3*n*-6, C20:4*n*-6, C20:5*n*-3, C22:5*n*-3 and C22:6*n*-3 with the addition of the macroalga, with or without feed enzymes, to the broiler diets. Also, the beneficial C18:3*n*-3 fatty acid was increased by combining *L. digitata* and commercial Rovabio® Excel AP compared to the control diet. The sum of SFA, MUFA and the *n*-6*/n*-3 PUFA ratio were decreased by *L. digitata*, regardless the addition of exogenous enzymes. β-carotene was enhanced by *L. digitata*, individually or combined with CAZymes, being also responsible for a positive increase in total pigments. Macrominerals, in particular phosphorous and sulphur, were increased in the liver of broilers fed *L. digitata* individually relative to the control. For microminerals, copper, iron and the correspondent sum were consistently elevated in the liver of broilers fed *L. digitata*, individually or combined with exogenous CAZymes. The powerful discriminant analysis tool based on the hepatic characterization revealed a good separation between the control group and *L. digitata* diets but failed to discriminate the addition of feed enzymes.

**Conclusions:**

Overall, this study highlights the value of *L. digitata* as a feed ingredient for the poultry industry. Moreover, we can conclude that the effect of *L. digitata* overpowers the effect of feed enzymes, both the Rovabio® Excel AP and the alginate lyase. Having in mind the negative effects observed on birds’ performance, our main recommendation at this stage is to restraint *L. digitata* incorporation level in forthcoming nutritional studies.

## Background

Chicken meat is considered a popular source of high-quality protein from animal origin for human consumption all over the world [1, 2]. But for the poultry industry, there is an increasing need to identify alternative feed ingredients that do not compete with foods for humans. Macroalgae, commonly known as seaweeds, might provide such a natural resource, but there is limited information concerning its value as an animal feed. Moreover, health-conscious consumers demand products with a high nutritional value to maintain health and well-being [2].

Macroalgae include a large number of diverse organisms, probably exceeding more than 25,000 species [3] of macroscopic, multicellular and marine nature [4]. Macroalgae belong to distinct and relatively unrelated eukaryotic lineages corresponding to taxonomically distant groups, often called green (*Chlorophyceae*), red (*Rhodophyceae*) and brown (*Phaeophyceae*) macroalgae. The nutritional composition of macroalgae, although highly variable with species, growing region, harvesting season [5, 5] and cultivation requirements, includes many vitamins, minerals, pigments and antioxidants, carbohydrates and high-quality proteins [6, 7]. Carbohydrates cover a high percentage of macroalgae biomass that may be ranged from 4 to 76% of dry matter (DM) [8], whereas lipids occur only in small amounts (< 5% of DM) with values reaching 1.13% of DM in brown macroalgae [9]. Notwithstanding, the lipid profile of macroalgae is typically enriched in monounsaturated (MUFA) and polyunsaturated (PUFA) fatty acids [10, 11] with well-known beneficial properties for human health [12, 13].

However, macroalgae contain recalcitrant polysaccharides in cell walls with anti-nutritional effects for monogastric animals, which negatively impacts on feed digestion and absorption efficiency by trapping valuable nutritional compounds [14, 15]. To overcome this problem, the dietary inclusion of specific Carbohydrate-Active enZymes (CAZymes) could allow the disruption of recalcitrant macroalga cell walls, with an increase in nutrients bioavailability. CAZymes are produced by microorganisms and are complex enzymes, in which the catalytic module(s) is (are) appended to one or more non-catalytic carbohydrate binding modules (CBM) [16]. According to the circumstances, the application of CAZymes for macroalgae biomass might represent a good strategy to value the nutritional compounds of cereal-based diets for monogastrics. In the pursuit of degrading macroalgae cell wall, it has been already shown that the in vitro addition of a single CAZyme, i.e. alginate lyase*,* among other benefits, improves lipid yield extraction and sugar recovery in *Laminaria digitata* (*L. digitata*), a typical example of a brown seaweed [17].

In view of these findings, the present study aimed to test if the dietary supplementation with feed enzymes, which included a commercially available Rovabio® Excel AP or an individual alginate lyase recently reported by Costa et al. [17], would improve nutrient availability from *L. digitata* incorporated at a high level in the diet (15%) and, therefore, promote birds´ growth performance and ameliorate general metabolic state. For the latter, the effects of dietary *L. digitata*, with or without feed carbohydrases, on plasma biochemical markers and liver lipid composition of broilers, covering also antioxidants deposition and mineral profile, were evaluated.

## Results

### Feed intake and broilers’ growth performance

Table 1 presents the results on feed intake and broilers’ growth performance. Diets had no impact on feed intake (*p* > 0.05). Final body weight was higher in broilers fed the control diet than those fed LA and LAE diets (*p* = 0.011). The feed conversion ratio (FCR) (*p* = 0.012) values were higher and the average daily gain (ADG) (*p* = 0.039) values were lower in broilers fed the LA diet than in those fed the control. However, ADG values did not differ (*p* > 0.05) between LAR and LAE diets and the control. Broilers´ mortality during the experimental period was low (2.5%) (data not shown), since only two animals fed the LAR diet and one fed the LAE diet had severe diarrhoea leading to death.

**Table 1:** Effect of experimental diets on growth performance parameters of broilers

Item	Control	LA	LAR	LAE	SEM	***p***-value
Initial weight, g	809.0	759.1	741.5	739.1	28.62	0.294
Final weight, g	1823^a^	1631^b^	1706^ab^	1644^b^	42.4	0.011
ADG, g/d	78.9^a^	67.6^b^	74.8^ab^	70.4^ab^	2.82	0.039
ADFI, g/pen	130	126	125	127	3.1	0.523
FCR	1.70^b^	1.89^a^	1.82^ab^	1.81^ab^	0.038	0.012

The broilers were fed: 1) a corn-soybean basal diet (Control); 2) the basal diet plus 15% of *L. digitata* (LA); 3) the basal diet plus 15% of *L. digitata* supplemented with 0.005% of Rovabio® Excel AP (LAR); and 4) the basal diet plus 15% of *L. digitata* supplemented with 0.01% of recombinant CAZyme (LAE)

*SEM* standard error of the mean, *ADG* average daily weight gain, *ADFI* average daily feed intake, *FCR* feed conversion ratio

^a.b^Different superscripts within a row indicate a significant difference (*p* <  0.05)

### Plasma metabolites

The plasma biochemical markers of broilers fed 15% of *L. digitata*, supplemented or not with CAZymes, are presented in Table 2. Total lipids (*p* = 0.001), triacylglycerols (TAG) (*p* <  0.001), VLDL-cholesterol (*p* <  0.001), total cholesterol/HDL-C ratio (*p* <  0.001) and total protein (*p* <  0.001) were consistently increased by exogenous CAZymes, both Rovabio® Excel AP and alginate lyase, relative to control and *Laminaria* diets. Total cholesterol (*p* = 0.037) and LDL-cholesterol (*p* = 0.004) were reduced by *L. digitata*, only when fed individually. Glucose was increased in *L. digitata* fed broilers (*p* = 0.001), regardless the addition of feed enzymes. The lowest value of urea was observed in *L. digitata* fed broilers (*p* <  0.001). Regarding the hepatic function, *L. digitata* reduced AST values with or without feed enzymes (*p* <  0.001). Conversely, the highest values of ALP (*p* <  0.001) and GGT (*p* <  0.001) were found in broilers fed the combination between *L. digitata* and alginate lyase in relation to the other dietary treatments. ALT (*p* = 0.079) was not affected by diets.

**Table 2:** Effect of experimental diets on plasma metabolites of broilers

Item	Control	LA	LAR	LAE	SEM	***p***-value
***Plasma metabolites***						
Total lipids (mg/L)^1^	3940^ab^	3810^b^	4100^a^	4040^a^	4.8	0.001
TAG (mg/L)	323^b^	348^b^	427^a^	416^a^	1.4	< 0.001
Total cholesterol (mg/L)	1060^a^	980^b^	1070^a^	1060^a^	2.4	0.037
HDL-cholesterol (mg/L)	824	774	793	783	1.6	0.157
LDL-cholesterol (mg/L)	173^a^	143^b^	170^a^	171^a^	0.6	0.004
VLDL-cholesterol (mg/L)^2^	64.6^b^	69.6^b^	85.4^a^	83.2^a^	0.28	< 0.001
Total cholesterol/HDL-C	1.28^b^	1.27^b^	1.35^a^	1.36^a^	0.012	< 0.001
Glucose (mg/L)	2390^b^	2500^a^	2470^a^	2480^a^	1.9	0.001
Urea (mg/L)	27.8^b^	22.5^c^	33.4^a^	31.6^ab^	0.12	< 0.001
Creatinine (mg/L)	< 0.010	< 0.010	< 0.010	< 0.010	–	–
Total protein (g/L)	22.3^b^	21.3^b^	24.7^a^	24.9^a^	0.03	< 0.001
*Plasma hepatic markers*						
ALT (U/L)	1.40	1.00	1.00	1.13	0.128	0.079
AST (U/L)	436^a^	209^b^	207^b^	184^b^	10.7	< 0.001
ALP (U/L)	1548^b^	1443^c^	1590^b^	2078^a^	23.6	< 0.001
GGT (U/L)	16.9^c^	28.4^b^	20.3^c^	34.0^a^	0.96	< 0.001

The broilers were fed: 1) a corn-soybean basal diet (Control); 2) the basal diet plus 15% of *L. digitata* (LA); 3) the basal diet plus 15% of *L. digitata* supplemented with 0.005% of Rovabio® Excel AP (LAR); and 4) the basal diet plus 15% of *L. digitata* supplemented with 0.01% of recombinant CAZyme (LAE)

*SEM* standard error of the mean, *TAG* triacylglycerols, *HDL* high-density lipoproteins, *LDL* low-density lipoproteins, *VLDL* very low-density lipoproteins, *ALT* alanine aminotransferase (EC 2.6.1.2), *AST* aspartate aminotransferase (EC. 2.6.1.1), *ALP* alkaline phosphatase (EC 3.1.3.1), *GGT* gamma-glutamyltransferase (EC 2.3.2.13)

^1^Total lipids = [total cholesterol] × 1.12 + [TAG] × 1.33 + 148, as described by Covaci et al. [18]

^2^VLDL-cholesterol = 1/5 [TAG], as described by Friedewald et al. [19]

^a.b,c^Different superscripts within a row indicate a significant difference (*p* <  0.05)

### Hepatic total lipids, cholesterol and fatty acid profile

The effects of *L. digitata,* individually or in combination with CAZymes, on hepatic total lipids, cholesterol content and fatty acid profile of broilers are shown in Table 3. Major variations were found for fatty acids across dietary treatments. The experimental diets had no impact on total lipids (*p* = 0.114) and total cholesterol (*p* = 0.090). The most prevalent fatty acids were C18:0 (26.6–25.4%), C18:2*n*-6 (24.8–21.8%), C20:4*n*-6 (17.3–13.8%), C16:0 (16.8–12.7%) and C18:1*c*9 (9.51–5.62% of total FAME). The percentage of C14:0 (*p =* 0.001), C16:0 (*p <* 0.001), C16:1*c*9 (*p <* 0.001), C18:1*c*9 (*p =* 0.001) and C20:3*n*-6 (*p <* 0.001) were reduced by *L. digitata*, regardless the addition of feed enzymes. On the contrary, C15:0 (*p <* 0.001), C17:0 (*p <* 0.001), C18:1*c*11 (*p <* 0.001), C18:2*n*-6 (*p =* 0.003), C18:3*n*-6 (*p =* 0.001), C20:0 (*p <* 0.001), C20:4*n*-6 (*p =* 0.001), C20:5*n*-3 (*p <* 0.001), C22:0 (*p <* 0.001), C22:5*n*-3 (*p <* 0.001) and C22:6*n*-3 (*p <* 0.001) were increased by the addition of macroalga with or without feed enzymes. C18:0 (*p =* 0.012) reached the highest percentage in broilers fed only *L. digitata*. The proportion of C18:3*n*-3 (*p =* 0.026) was increased by the combination of *L. digitata* and Rovabio® Excel AP when compared to the control diet. The sum of SFA (*p <* 0.001), MUFA (*p =* 0.001) and the n-6/n-3 PUFA ratio (*p <* 0.001) were decreased by *L. digitata* with or without feed enzymes. An inverse trend was observed for total PUFA (*p <* 0.001), *n*-6 PUFA (*p <* 0.001), *n*-3 PUFA (*p <* 0.001), and PUFA/SFA ratio.

**Table 3:** Effect of experimental diets on hepatic total lipids, total cholesterol and fatty acid composition of broilers

Item	Control	LA	LAR	LAE	SEM	***p***-value
***Total lipids, g/100 g***	3.14	2.90	2.77	3.03	0.339	0.114
***Total cholesterol, mg/g***	1.84	2.11	1.90	2.02	0.080	0.090
***FA composition, g/100 g FA***						
C10:0	0.006	0.014	0.007	0.007	0.0026	0.136
C12:0	0.001	0.002	0.001	0.002	0.0012	0.868
C14:0	0.155^a^	0.076^b^	0.080^b^	0.090^b^	0.0092	0.001
C15:0	0.045^b^	0.061^a^	0.062^a^	0.062^a^	0.0021	< 0.001
C16:0	16.8^a^	12.7^b^	13.0^b^	13.0^b^	0.30	< 0.001
C16:1*c*7	0.268^ab^	0.233^b^	0.235^b^	0.308^a^	0.0183	0.021
C16:1*c*9	0.167^a^	0.072^b^	0.072^b^	0.085^b^	0.0101	< 0.001
C17:0	0.280^b^	0.406^a^	0.404^a^	0.389^a^	0.0096	< 0.001
C17:1*c*9	0.002	0.001	0.000	0.003	0.0012	0.268
C18:0	25.4^b^	26.6^a^	26.0^ab^	25.9^ab^	0.25	0.012
C18:1*c*9	9.51^a^	5.73^b^	5.62^b^	6.16^b^	0.417	0.001
C18:1*c*11	0.758^b^	0.893^a^	0.909^a^	0.873^a^	0.0219	< 0.001
C18:2*n*-6	21.8^b^	24.7^a^	24.5^a^	24.8^a^	0.44	0.003
C18:3*n*-6	0.051^b^	0.062^a^	0.065^a^	0.062^a^	0.0023	0.001
C18:2*t*9*t*12	0.186	0.166	0.179	0.183	0.0149	0.790
C18:3*n*-3	0.075^b^	0.091^ab^	0.094^a^	0.091^ab^	0.0046	0.026
C18:4*n*-3	0.020	0.025	0.019	0.020	0.0027	0.387
C20:0	0.084^b^	0.149^a^	0.150^a^	0.126^a^	0.0090	< 0.001
C20:1*c*11	0.215	0.202	0.202	0.196	0.0126	0.751
C20:2*n*-6	1.33	1.29	1.34	1.32	0.093	0.981
C20:3*n*-6	1.93^a^	0.87^b^	0.84^b^	1.00^b^	0.068	< 0.001
C20:4*n*-6	13.8^b^	17.2^a^	17.3^a^	16.7^a^	0.40	0.001
C20:3*n*-3	0.018	0.023	0.019	0.019	0.0050	0.897
C20:5*n*-3	0.026^b^	0.105^a^	0.093^a^	0.089^a^	0.0051	< 0.001
C22:0	0.037^b^	0.059^a^	0.059^a^	0.054^a^	0.0033	< 0.001
C22:1*n*-9	0.017	0.015	0.000	0.006	0.0051	0.240
C22:2*n*-6	0.000	0.000	0.000	0.002	0.0007	0.099
C22:5*n*-3	0.250^b^	0.834^a^	0.876^a^	0.783^a^	0.0411	< 0.001
C22:6*n*-3	0.68^b^	2.50^a^	2.72^a^	2.73^a^	0.154	< 0.001
Others	6.08^a^	4.88^b^	5.28^ab^	4.94^b^	0.283	0.019
***Partial sums of FA, g/100 g FA***						
SFA^1^	42.8^a^	40.1^b^	39.7^b^	39.6^b^	0.43	< 0.001
MUFA^2^	10.91^a^	7.13^b^	7.03^b^	7.61^b^	0.43	0.001
PUFA^3^	40.2^b^	47.9^a^	48.0^a^	47.8^a^	0.57	< 0.001
*n*-6 PUFA^4^	38.9^b^	44.1^a^	44.0^a^	43.9^a^	0.53	< 0.001
*n*-3 PUFA^5^	1.07^b^	3.58^a^	3.82^a^	3.73^a^	0.169	< 0.001
*Ratios of FA*						
PUFA/SFA	0.94^b^	1.20^a^	1.21^a^	1.21^a^	0.021	< 0.001
*n*-6/*n*-3	38.5^a^	12.9^b^	11.7^b^	12.0^b^	1.55	< 0.001

The broilers were fed: 1) a corn-soybean basal diet (Control); 2) the basal diet plus 15% of *L. digitata* (LA); 3) the basal diet plus 15% of *L. digitata* supplemented with 0.005% of Rovabio® Excel AP (LAR); and 4) the basal diet plus 15% of *L. digitata* supplemented with 0.01% of recombinant CAZyme (LAE)

*SEM* standard error of the mean, *FA* fatty acids, *SFA* saturated fatty acids, *MUFA* monounsaturated fatty acids, *PUFA* polyunsaturated fatty acids

^1^Sum (C10:0, C12:0, C14:0, C15:0, C16:0, C17:0, C18:0, C20:0, C22:0)

^2^Sum (C16:1*c*7, C16:1*c*9, C17:1*c*9, C18:1*c*9, C18:1*c*11, C20:1*c*11, C22:1*n*-9)

^3^Sum (C18:2*n*-6, C18:2*t*9*t*12, C18:3*n*-6, C18:3*n*-3, C18:4*n*-3, C20:2*n*-6, C20:3*n*-6, C20:4*n*-6, C20:3*n*-3, C20:5*n*-3, C22:5*n*-3, C22:6*n*-3)

^4^Sum (C18:2*n*-6, C18:3*n*-6, C20:2*n*-6, C20:3*n*-6, C20:4*n*-6)

^5^Sum (C18:3*n*-3, C18:4*n*-3, C20:3*n*-3, C20:5*n*-3, C22:5*n*-3, C22:6*n*-3)

^a,b^Different superscripts within a row indicate a significant difference (*p* <  0.05)

### Hepatic vitamin E and pigments

Hepatic tocopherols and pigments of broilers fed *L. digitata*, supplemented or not with CAZymes, are shown in Table 4. α-Tocopherol (*p* = 0.030) was reduced by *L. digitata* and Rovabio® Excel AP relative to the control diet. Similarly, broilers fed macroalga showed reduced γ-tocopherol content, regardless the presence of CAZymes (*p* <  0.001). In contrast, β-carotene (*p =* 0.0008) was increased by *L. digitata*, individually or combined, compared to the control diet influencing also, and in the same manner, the sum of total chlorophylls and carotenoids (*p =* 0.001). The chlorophyll a (*p* = 0.033) reached the highest content in broilers fed *L. digitata* plus alginate lyase whereas total chlorophylls (*p* = 0.065), including chlorophyll b (*p* = 0.096) did not change across diets.

**Table 4:** Effect of experimental diets on hepatic vitamin E homologues and pigments from broilers

Item	Control	LA	LAR	LAE	SEM	***p***-value
***Diterpene profile, μg/g***						
α-Tocopherol	22.3^a^	18.7^ab^	15.2^b^	20.3^ab^	1.63	0.030
γ-Tocopherol	0.526^a^	0.281^b^	0.175^b^	0.267^b^	0.0414	< 0.001
***Pigments, μg/100 g***						
β-Carotene	22.0^b^	40.3^a^	40.8^a^	42.6^a^	0.04	< 0.001
Chlorophyll-*a*^1^	9.34^b^	14.3^ab^	17.0^ab^	20.0^a^	2.52	0.033
Chlorophyll-*b*^2^	16.8	22.1	26.6	31.7	4.22	0.096
Total chlorophylls^3^	26.1	36.4	43.6	51.7	6.72	0.065
Total carotenoids^4^	170^b^	227^a^	208^ab^	222^a^	10.0	0.001
Total chlorophylls+carotenoids^5^	196^b^	263^a^	251^a^	273^a^	13.2	0.001

The broilers were fed: 1) a corn-soybean basal diet (Control); 2) the basal diet plus 15% of *L. digitata* (LA); 3) the basal diet plus 15% of *L. digitata* supplemented with 0.005% of Rovabio® Excel AP (LAR); and 4) the basal diet plus 15% of *L. digitata* supplemented with 0.01% of recombinant CAZyme (LAE)

*SEM* standard error of the mean

^1^Ca = 11.24 × A_662_–2.04 × A_645_

^2^Cb = 0.13 × A_645_–4.19 × A_662_

^3^Ca + b = 7.05 × A_662_ + 18.09 × A_645_

^4^Cx + c = (1000 × A_470_–1.90 × Ca - 63.14 × Cb) / 214

^5^(Ca + b) + (Cx + c)

^a.b,c^Different superscripts within a row indicate a significant difference (*p* <  0.05)

### Hepatic minerals and trace elements content

The hepatic content of minerals from broilers fed *L. digitata*, supplemented or not with CAZymes, are shown in Table 5. The sum of macrominerals (*p* = 0.015), as well as the elements phosphorous (*p* = 0.013) and sulphur (*p* = 0.006), are increased in broilers fed *L. digitata* individually relative to the control. The same was observed for the sum of macrominerals and microminerals (*p* = 0.007). The levels found for copper (*p* <  0.001), iron (*p* <  0.001) and manganese (*p* <  0.001) were consistently higher in broilers fed *L. digitata*, individually or combined with exogenous CAZymes. Zinc level was higher in broilers fed *L. digitata* combined with the alginate lyase, intermediate in broilers fed *L. digitata* individually or combined with Rovabio® Excel AP, and lower in the control (*p* = 0.044).

**Table 5:** Effect of experimental diets on the mineral content of liver from broilers

Item	Control	LA	LAR	LAE	SEM	***p***-value
***Macrominerals, mg/100 g***						
Calcium	19.3	20.0	19.7	19.6	0.51	0.837
Magnesium	24.2	24.5	23.9	24.2	0.57	0.878
Potassium	442	462	444	457	8.4	0.250
Phosphorous	296^b^	321^a^	308^ab^	308^ab^	5.0	0.013
Sodium	79.2	84.6	81.8	79.0	2.16	0.242
Sulphur	194^b^	216^a^	205^ab^	210^ab^	4.2	0.006
Total	1054^b^	1128^a^	1082^ab^	1097^ab^	15.4	0.015
***Microminerals, mg/100 g***						
Copper	0.325^b^	0.417^a^	0.409^a^	0.406^a^	0.0092	< 0.001
Iron	9.7^c^	15.5^b^	15.0^b^	19.5^a^	0.83	< 0.001
Manganese	0.389^b^	0.536^a^	0.526^a^	0.550^a^	0.0191	< 0.001
Zinc	3.09^b^	3.17^ab^	3.38^ab^	3.70^a^	0.157	0.044
Total	13.5^c^	19.6^b^	19.3^b^	24.1^a^	0.85	< 0.001
***Total macro- and microminerals***	1068^b^	1148^a^	1101^ab^	1122^ab^	15.4	0.007

The broilers were fed: 1) a corn-soybean basal diet (Control); 2) the basal diet plus 15% of *L. digitata* (LA); 3) the basal diet plus 15% of *L. digitata* supplemented with 0.005% of Rovabio® Excel AP (LAR); and 4) the basal diet plus 15% of *L. digitata* supplemented with 0.01% of recombinant CAZyme (LAE)

*SEM* standard error of the mean

^a.b,c^Different superscripts within a row indicate a significant difference (*p* < 0.05)

### Principal component analysis (PCA)

**Fig. 1:**
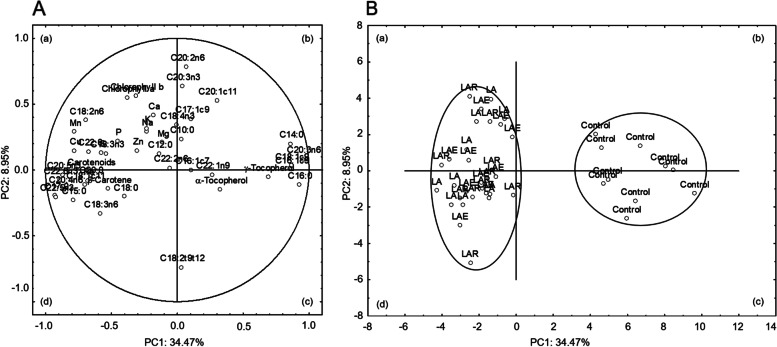
Loading plot of the 1st and 2nd principal components (PC) of the pooled data (**A**) and component score vectors (**B**) using hepatic parameters from broilers fed *L. digitata*, with or without the commercially available Rovabio® Excel AP or an individual alginate lyase. The broilers were fed: 1) a corn-soybean basal diet (Control); 2) the basal diet plus 15% of *L. digitata* (LA); 3) the basal diet plus 15% of *L. digitata* supplemented with 0.005% of Rovabio® Excel AP (LAR); and 4) the basal diet plus 15% of *L. digitata* supplemented with 0.01% of recombinant CAZyme (LAE)

The principal component analysis was carried out to assess the relationship among fatty acids, pigments and minerals in the liver of broilers fed the experimental diets, showing two dimensional data variability. The first two principal components justified 43.42% of the total variance, being PC1 accountable for 34.47% and PC2 accountable for 8.95%. As total variance explained by the first two PC is close to 50%, the projection of those parameters in the plane defined by these two PC is shown in Fig. 1A. The loadings for the first two PC obtained for each parameter are shown in Table 6. The variables that have high loadings (positive or negative) are the ones that mostly contribute to each PC. A positive loading means that a variable correlates positively with the PC, whereas a negative loading indicates a negative correlation. Overall, PC1 was mainly characterized by C20:3*n*-6 (0.95), C16:0 (0.92), C14:0 (0.86) and C16:1*c*9 (0.86) fatty acids on the right, and by C17:0 (− 0.93), C22:5*n*-3 (− 0.92), C22:6*n*-3 (− 0.89), C20:5*n*-3 (− 0.87), C20:4*n*-6 (− 0.86), C15:0 (− 0.79) and manganese (− 0.78) on the left. The PC2 clearly distinguished C20:2*n*-6 (0.79), C20:3*n*-3 (0.64), chlorophyll b (0.56), chlorophyll a (0.55) and C20:1*c*11 (0.53) located in the upper part from C18:2*t*9*t*12 (− 0.74) located in the lower part of the graphic (Fig. 1A). The PCA model revealed a good separation between the control group and *L. digitata* based diets (Fig. 1B). The control group was confined to quadrants *b* and *c* and clearly discriminated from the others. Macroalga-based dietary groups supplemented or not with exogenous enzymes were located more dispersed in quadrants *a* and *d* with no possible discrimination on the addition of feed enzymes (LA, LAR and LAE dietary groups).

**Table 6:** Loadings for the first two principal components (PC) in the liver

Variables	PC 1	PC 2
C10:0	0.03	0.24
C12:0	−0.14	0.12
C14:0	0.86	0.20
C15:0	−0.79	−0.23
C16:0	0.92	−0.11
C16:1*c*7	0.10	0.00
C16:1*c*9	0.86	0.01
C17:0	−0.93	− 0.19
C17:1c9	0.10	0.39
C18:0	−0.40	− 0.20
C18:1*c*9	0.86	0.03
C18:1*c*11	−0.70	−0.11
C18:2*n*-6	−0.70	0.38
C18:3*n*-6	−0.59	−0.33
C18:2*t*9*t*12	0.03	−0.74
C18:3*n*-3	−0.54	0.13
C18:4*n*-3	−0.01	0.34
C20:0	−0.66	−0.07
C20:1*c*11	0.30	0.53
C20:2*n*-6	0.07	0.79
C20:3*n*-6	0.95	0.08
C20:4*n*-6	−0.86	−0.14
C20:3*n*-3	0.04	0.64
C20:5*n*-3	−0.87	−0.03
C22:0	−0.67	0.14
C22:1*n*-9	0.26	−0.03
C22:2*n*-6	−0.06	0.02
C22:5*n*-3	−0.92	−0.20
C22:6*n*-3	−0.89	−0.08
β-Carotene	−0.53	−0.14
α-Tocopherol	0.32	−0.14
γ-Tocopherol	0.69	−0.05
Chlorophyll-*a*	−0.38	0.55
Chlorophyll-*b*	−0.32	0.56
Carotenoids	−0.66	−0.01
Na	−0.23	0.29
K	−0.24	0.32
Ca	−0.18	0.42
Mg	−0.12	0.21
P	−0.46	0.22
S	−0.58	0.13
Cu	−0.78	0.15
Zn	−0.31	0.15
Mn	−0.78	0.29
Fe	−0.68	−0.08

## Discussion

Macroalga cell walls contain a wide variety of complex carbohydrates, such as the polysaccharides, alginate and fucoidan. The different ability of monogastric species to digest macroalga cell walls and, thus, the level of macroalga in the diet [5] naturally impacts animal growth. In the present study, the dietary incorporation of 15% of *L. digitata* had a negative impact on broilers productive parameters by reducing final body weight and ADG and increasing FCR. Similarly, when a 20 and 30% brown algae mixture containing 8 different seaweed species was added to broiler chicks’ diet, it was found a decrease on final body weight, whereas a 10% inclusion caused the opposite effect [20]. Ahmed et al. [5] reported no effects on the growth performance of broiler chickens by the dietary supplementation with 5 g/kg of fermented kelp. Furthermore, 0.1, 0.5 and 1.0% of *Laminaria japonica* (*L. japonica*) supplementation had no adverse effect on the overall growth performance of growing ducks [21]. In fact, Ventura et al. [22] even suggested that the dietary inclusion of macroalgae as a feed ingredient for poultry should be limited to 10% of feed due to the high content of indigestible polysaccharides present in seaweeds. In addition, the type of alga (e.g. nutritive value) [23] and the growth stage of the chicks are factors affecting the efficiency of macroalga as feed ingredient.

Brown macroalgae, including other species than *L. digitata,* such as *Undaria pinnatifida*, *Hizikia fusiformis* [24] and algae mixture composed by *Laminaria hyperborea*, *Macrocystis pyrifera*, *Lessonia nigrescens* and *Ascophyllum nodosum* [25], have also been used as feed supplements for poultry diets and might display relevant functions as prebiotics, thus promoting animal growth. Indeed, purified polymannuronate, a polymer of (1–4) linked β-D-mannuronic acid that is part of alginate extracted from this alga mixture, changes caecal bacterial diversity, causing a rise on lactic acid bacteria and a decrease of *Escherichia coli* counts, and displays also immunomodulatory characteristics, as the ability to promote Ig M increase, when fed to broilers at 0.4 to 0.5% feed. This prebiotic activity, together with the consequent alteration of gut fermentation probably contributed to the increase of ADG and decrease of FCR in chicks [25], contrasting with our own results. Data on the relationship between the level of inclusion of macroalga and G:F in poultry show that this relationship is once more dependent on both algae and birds species. In addition, feeding brown macroalga *Hizikia fusiformis* by-products [24] and a purified polymannuronate from a brown alga mix [25] at 0.4 to 0.5% feed led to an increase of feed efficiency. It also should be underlined that the percentage of *L. digitata* incorporation in the diet and the duration of the experimental period impact broilers’ growth performance parameters [26]. In the present study, similarly to what was previously reported for polymannuronate polymer of alginate used as a feed supplement for broilers [25], the dietary supplementation with the in vitro selected alginate lyase [17] was expected to release bioactive polysaccharides from *L. digitata* cell wall, such as alginate, with prebiotic activity and, consequently, improve broiler performance. However, the effect of feed enzymes was residual and did not prevent the impairment of animal growth performance caused by incorporating high levels of seaweed in broilers´ diet, even though enzyme supplementation slightly counterbalanced the negative effects on body weight and ADG found with LA treatment. At the present algae inclusion level, the high mineral and non-starch polysaccharide contents of *L. digitata* were probably the cause of broiler growth impairment, as suggested in a recent study [27] where an increase of FCR was observed in broilers fed 10% of silage or silage residue of *Saccharina latissima*.

Plasma levels of biochemistry parameters provide valuable information on the physiological conditions of the animal of interest [28]. The lipid profile was greatly influenced by diets. Our data show that total cholesterol and LDL-cholesterol were reduced by *L. digitata*, when fed individually. Corroborating these data, LDL concentration was low in 0.1 and 1.0% of *L. japonica* supplemented groups using ducks [22].

Conversely, total lipids, TAG, VLDL-cholesterol, and total cholesterol/HDL-C ratio were increased by exogenous CAZymes, both Rovabio® Excel AP and alginate lyase. The raise on VLDL cholesterol transport stimulated by this macroalga in combination with CAZymes was not countered by HDL reverse cholesterol, enhancing total cholesterol/HDL-cholesterol ratio. The present study highlights cholesterol- and lipid-lowering properties of individual *L. digitata*, although the mechanism of this hypocholesterolaemic action is still unclear. As previously observed for some microalga species [29], this improved effect on lipemia upon supplementation with *L. digitata* can be attributed to a decrease in intestinal tract fat absorption [26]. Glucose was slightly increased in *L. digitata* fed broilers, independently of the addition of feed enzymes. Still concerning plasma reference values, the lowest level of urea was observed in *L. digitata* fed broilers suggesting unaffected renal function by the macroalga addition [26, 29]. Regarding the hepatic markers, the variations observed for the enzymatic activities of ALP and GGT are still within the reference figures for birds [30, 30] and therefore, are devoid of clinical physiological relevance. Various algae-derived bioactive components, such as lipids, antioxidants, pigments, vitamins and polysaccharides are recognized as beneficial to animal and human health. Although the available literature on the effects of *L. digitata* as a feed ingredient in poultry is scarce, the divergence observed in response to hepatic and renal metabolites in different trials could be partly attributed to the dose and origin of macroalga, as well as duration of the experimental period and settings.

The central organ for cholesterol synthesis and fatty acid oxidation is the liver. Moreover, de novo lipogenesis occurs basically in liver and adipose tissue [31]. Total lipids and cholesterol concentration in the liver were unaffected by 15% of *L. digitata* dietary incorporation, nor by the supplementation with exogenous CAZymes. Major changes were found for *n*-3 fatty acids, such as eicosapentaenoic acid (*EPA,* C20:5n-3), docosapentaenoic acid (DPA, C22:5*n*-3) and docosahexaenoic acid (DHA, C22:6*n*-3), which were increased by the addition of the *L. digitata*, with or without feed enzymes. Also, the essential alpha-linolenic acid (ALA, C18:3*n*-3) was increased by the combination of *L. digitata* and commercial Rovabio® Excel AP relative to the control diet. In fresh duck meat, the concentration of *n*-3 fatty acids was also elevated upon 1.0% of *L. japonica* supplementation [22] suggesting that this level of dietary supplementation can be used as a potential alternative to antibiotics in ducks’ production [22]. It was proposed that the higher concentration of *n*-3 PUFA in *L. japonica* dietary group was due to the presence of phospholipids and glycolipids [22]. Our data agree with findings reported by Islam et al. [22], Pulz and Gross [32] and Plaza et al. [33] and who found that seaweed contains substantial amounts of *n*-3 fatty acids. These are substances of particular interest in animal feeding due to their anti-microbial and antioxidant properties, as well as their biofortification ability of animal products [34]. Moreover, the enrichment in *n*-3 PUFA in the liver has been linked to downregulation of PUFA oxidation-related genes expression, mitigated lipid peroxidation and increased antioxidant properties [35].

We also looked for the impact of *L. digitata* dietary incorporation, with or without CAZymes, on hepatic levels of tocopherols and pigments. Vitamin E is the major free radical chain terminator in the lipophilic environment [36]. Among the vitamin E compounds, α-tocopherol was the major vitamin E homologue in all dietary groups, whereas γ-tocopherol was the minor, which strongly agree with diets composition. Brown algae as *Laminaria* spp., and other species have a high content of vitamin E [37]. The combination of *L. digitata* and Rovabio® Excel AP reduced α-tocopherol content whereas the macroalga per se reduced γ-tocopherol, relative to the control. Both findings are not in according with diets composition. On the contrary, β-carotene, a precursor of vitamin A, was increased by *L. digitata*, aligning well with the fact that *L. digitata* is β-carotene enriched [38]. The raise of β-carotene and total chlorophylls and carotenoids contents in the liver is a key indicator of their correspondent dietary bioavailability. Chlorophylls and carotenoids are natural lipophilic pigments that have been studied for their role in maintaining antioxidant homeostasis [39], highly relevant for animals and human health [40]. At this stage, it should also be emphasized that all of the main constituents of the macroalgal biomass vary not only between species, but also with location, season and maturity of the macroalga [41].

In general, macroalgae contain high mineral content [6, 42]. This is considered a positive trait with a favourable impact in animal feeds [6], due to the high importance of minerals in many organic functions, such as cellular metabolism (e.g. iodine) and osmotic regulation (e.g. sodium). Besides several elements which are ubiquitous in many biological matrices, such as sodium, magnesium, potassium and calcium, the mineral component of macroalgae is very often rich in bromine and iodine [9]. These elements are found at much lower levels in other potential sources of feed ingredients. The impact of *L. digitata* experimental diets on the mineral content of liver from broilers was also exploited. Unfortunately, we did not characterize hepatic iodine. The sum of macrominerals as well as phosphorous and sulphur were increased in broilers fed only *L. digitata*, not supporting the amounts quantified for these elements in dietary regimens. Regarding microminerals, the same was observed for copper, iron and manganese, regardless the addition of feed enzymes. Zinc was highest in broilers fed *L. digitata* combined with the alginate lyase, intermediate in broilers fed *L. digitata* individually or combined with Rovabio® Excel AP, and lowest in broilers fed control diet. Both microminerals and macrominerals quantified are not very far from the reference values collected by Costa et al. [9] for *Laminaria* sp. Zinc, manganese and copper display a key co-factor role of antioxidant enzymes, such as superoxide dismutase [43]. As far it concerns to iron, this metallic chemical element is known to be essential to life, but it is poorly soluble in biological fluids and toxic when exists in excess. Iron plays a central role in generating harmful oxygen species. Its redox cycling can promote the Fenton reaction in which the potent oxidant hydroxyl radical is produced. Normally iron is transported, used, and stored in specific proteins (transferrin, ferritin, haem proteins, among others) [44, 45]. Overall, hepatic variations of copper, iron, manganese and zinc levels do not concur with diets composition. But most importantly, the changes herein observed across diets suggest that *L. digitata* provided the above mentioned macro- and microminerals to birds’ health.

## Conclusion

Summing up, our data indicate that *L. digitata* incorporated as a feed ingredient (15%) to poultry diets decreases systemic lipemia, suggesting a hypocholesterolaemic action, and improves hepatic fatty acid composition, by increasing the protective *n*-3 PUFA. As far as pigments concern, *L. digitata* increased the contents of β-carotene, influencing also the sum of total chlorophylls and carotenoids. The mineral composition for elements, such as copper, iron, manganese and zinc followed the same positive trend. Apart from minor variations, the supplementation of diets with the exogenous CAZymes, either the commercial Rovabio® Excel AP or the alginate lyase, was found as residual. That is why the powerful discriminant analysis tool based on the hepatic characterization revealed a good separation between the control group and *L. digitata* diets but failed to discriminate the addition of feed enzymes. Although the main results suggest the viability of *L. digitata* as feedstock in poultry nutrition, the negative effects observed on birds’ growth performance make *L. digitata* hard to be accepted as a feed ingredient with an inclusion rate as high as 15%. This aspect cannot be overlooked in forthcoming nutritional studies in broilers, thereby a lower level of seaweed incorporation as an ingredient along with novel exogenous enzymes should be pursued.

## Methods

### Broilers management and experimental diets

All experimental procedures were carefully reviewed by the Ethics Commission of CIISA/FMV and the Animal Care Committee of National Veterinary Authority (Direção Geral de Alimentação e Veterinária, Portugal), according to specific guidelines of European Union legislation (2010/63/EU Directive) and following ARRIVE Guidelines for in vivo experiments.

A total of 120, 1-day-old male Ross 308 broiler chicks with an average body weight of 45.1 ± 0.23 g were housed in 40 wired-floor cages for 35 days, as previously described [30, 46]. Briefly, birds were raised under environmentally controlled conditions, with continuously monitored temperature and ventilation. Three birds were allocated per pen with 10 replicate pens per treatment, in order to reduce the number of animals used in the experiment (3R’s principle) and according to previous reports [26, 46], and submitted to an adaptation period of 21 days, in which they were fed a corn and soybean meal-based diet, followed by an experimental period of 14 days until the standard slaughter age of 35 days (finishing period), in which they received one of the 4 experimental diets: 1) a corn-soybean meal based diet (Control); 2) the control diet with 15% of *L. digitata* powder (Algolesko; Plobannalec-Lesconil, Brittany, France) (LA); 3) the control diet with 15% of *L. digitata* powder and supplemented with 0.005% of a commercial CAZyme mixture, Rovabio® Excel AP (Adisseo; Antony, France) (LAR); and 4) the control diet with 15% of *L. digitata* powder and supplemented with 0.01% of a recombinant CAZyme (LAE). The Rovabio® Excel AP contained β-xylanase and β-glucanase, whereas the recombinant CAZyme was an alginate lyase belonging to PL7 family and described by Costa et al. [17]. Diets were formulated to be isocaloric and isonitrogenous. The feed ingredients and chemical composition of *L. digitata* and experimental diets are presented in Tables 7 and 8.

**Table 7:** Ingredients and additives of the experimental diets (% as fed basis)

Dietary treatments				
Ingredients	Control	LA	LAR	LAE
Corn	50.4	32.6	32.6	32.6
Soybean meal	41.2	42.9	42.9	42.9
Sunflower oil	4.80	6.93	6.93	6.93
Sodium chloride	0.38	0.00	0.00	0.00
Calcium carbonate	1.10	0.90	0.90	0.90
Dicalcium phosphate	1.6	1.12	1.12	1.12
DL-Methionine	0.12	0.15	0.15	0.15
L-Lysine	0.00	0.00	0.00	0.00
Vitamin-mineral premix^a^	0.40	0.40	0.40	0.40
*Laminaria digitata* powder	–	15.0	15.0	15.0
Rovabio® Excel AP	–	–	0.005	–
Recombinant CAZyme	–	–	–	0.01

The broilers were fed: 1) a corn-soybean basal diet (Control); 2) the basal diet plus 15% of *L. digitata* (LA); 3) the basal diet plus 15% of *L. digitata* supplemented with 0.005% of Rovabio® Excel AP (LAR); and 4) the basal diet plus 15% of *L. digitata* supplemented with 0.01% of recombinant CAZyme (LAE)

^a^Premix provided per kg of diet: pantothenic acid 10 mg, vitamin D3 2400 IU, cyanocobalamin 0.02 mg, folic acid 1 mg, vitamin K3 2 mg, nicotinic acid 25 mg; vitamin B6 2 mg, vitamin A 10000 UI, vitamin B1 2 mg, vitamin E 30 mg, vitamin B2 4 mg, Cu 8 mg, Fe 50 mg, I 0.7 mg, Mn 60 mg, Se 0.18 mg, Zn 40 mg

**Table 8:** Chemical composition of *Laminaria digitata* and experimental diets

	Macroalga	Experimental Diets			
Item	*L. digitata*	Control	LA	LAR	LAE
*Energy, kcal ME/kg as dry matter*	3065	4178	4184	4209	4201
*Proximate composition, % as dry matter*					
Dry matter	90.8	89.8	89.8	90.1	90.1
Crude protein	4.85	23.0	23.7	23.5	23.3
Crude fat	1.31	8.28	9.95	10.1	10.2
Ash	17.4	6.50	7.40	7.50	7.60
*Estimated available limiting amino acid composition, % as fed basis*					
Arginine	–	1.54	1.54	1.54	1.54
Histidine	–	0.59	0.57	0.57	0.57
Isoleucine	–	1.12	1.12	1.12	1.12
Leucine	–	1.91	1.80	1.80	1.80
Lysine	–	1.23	1.24	1.24	1.24
Methionine	–	0.47	0.48	0.48	0.48
Phenylalanine	–	1.22	1.19	1.19	1.19
Threonine	–	0.85	0.83	0.83	0.83
Tryptophan	–	0.32	0.32	0.32	0.32
Valine	–	1.20	1.17	1.17	1.17
*Fatty acid profile, % total fatty acids*					
C14:0	5.12	0.088	0.206	0.207	0.214
C16:0	22.7	9.13	8.78	8.84	8.79
C16:1*c*9	2.95	0.114	0.174	0.175	0.175
C17:0	0.454	0.051	0.050	0.049	0.047
C17:1*c*9	0.581	0.026	0.036	0.038	0.039
C18:0	1.09	3.05	3.10	3.13	3.17
C18:1*c*9	19.3	27.5	26.5	26.4	27.2
C18:2*n*-6	8.32	56.4	56.8	56.8	56.1
C18:3*n*-3	5.14	0.888	0.935	0.932	0.919
C18:4*n*-3	5.84	0.005	0.143	0.149	0.149
C20:0	0.931	0.345	0.316	0.324	0.320
C20:4*n*-6	9.79	0.001	0.208	0.211	0.218
C20:5*n*-3	13.8	0.004	0.276	0.280	0.290
*Diterpene profile, μg/g*					
α-Tocopherol	38.2	71.4	92.2	85.8	81.7
α-Tocotrienol	n.d^†^	6.39	3.55	3.19	3.09
β-Tocopherol	0.180	1.07	1.10	0.929	0.960
γ-Tocopherol+β-tocotrienol	0.129	5.68	3.44	3.06	3.26
γ-Tocotrienol	n.d	6.13	3.85	3.25	3.23
δ-Tocopherol	n.d	1.11	0.759	0.597	0.730
*Pigments, μg/g*					
β-Carotene	7.34	0.846	3.30	3.49	2.87
Chlorophyll-*a*^a^	235	1.72	58.6	57.2	58.6
Chlorophyll-*b*^b^	4.40	0.566	1.01	0.855	1.21
Total chlorophylls^c^	239	2.29	59.6	58.01	59.8
Total carotenoids^d^	93.9	2.87	21.6	21.6	21.7
Total chlorophylls + Carotenoids^e^	333	5.16	81.2	79.6	81.5
*Mineral profile, mg/kg dry matter*					
Arsenic	40.3	n.d.	n.d.	n.d.	n.d.
Barium	5.94	n.d.	n.d.	n.d.	n.d.
Bromide	474	4.79	131	131	122
Cadmium	0.0716	n.d.	n.d.	n.d.	n.d.
Calcium	8819	28,128	17,327	18,392	17,530
Cobalt	n.d.	n.d.	n.d.	n.d.	n.d.
Copper	2.88	26.68	16.06	15.42	15.68
Iron	144	407	237	274	241
Lead	n.d.	n.d.	n.d.	n.d.	n.d.
Magnesium	5637	2648	3326	3466	3276
Manganese	5.42	218	154	171	160
Nickel	n.d.	n.d.	n.d.	n.d.	n.d.
Phosphorous	903	12,129	7647	7881	7673
Potassium	28,530	15,676	19,011	19,237	18,596
Sodium	22,627	3563	5807	6495	6077
Sulphur	7653	4012	4474	4664	4599
Vanadium	1.34	n.d.	n.d.	n.d.	n.d.
Zinc	28.1	233	147	168	145

The broilers were fed: 1) a corn-soybean basal diet (Control); 2) the basal diet plus 15% of *L. digitata* (LA); 3) the basal diet plus 15% of *L. digitata* supplemented with 0.005% of Rovabio® Excel AP (LAR); and 4) the basal diet plus 15% of *L. digitata* supplemented with 0.01% of recombinant CAZyme (LAE)

*SEM* standard error of the mean

*DM* dry matter, *ME* metabolized energy, *n.d.* not detected

^†^Co-eluted with α-tocopherol

^a^Ca = 11.24 × A_662_–2.04 × A_645_

^b^Cb = 0.13 × A_645_–4.19 × A_662_

^c^Ca + b = 7.05 × A_662_ + 18.09 × A_645_

^d^Cx + c = (1000 × A_470_–1.90 × Ca - 63.14 × Cb) / 214

^e^(Ca + b) + (Cx + c)

### Production of the recombinant CAZyme

Plasmids containing the genes encoding the recombinant alginate lyase were obtained as described by Costa et al. [17]. *Escherichia coli* (BL21) was transformed with the plasmids and grown to mid exponential phase (absorbance between 0.4 and 0.6, λ = 595 nm) on Luria-Bertani media at 37 °C, 200 rpm, with Kanamycin (50 mg/mL). The recombinant gene was expressed in an NZY auto-induction LB medium (Nzytech, Lisbon, Portugal) incubated overnight at 25 °C at 140 rpm. Afterwards, cells were submitted to ultrasonication and centrifugation and the protein extract (supernatant) was recovered. Finally, the extract was freeze-dried and included, in equal weight proportions, at a final level of 0.01% in the LAE diet.

### Chemical analysis of *L. digitata* and experimental diets

The chemical composition of *L. digitata* and experimental diets is presented in Table 2. The alga and feed DM, crude protein, ash, crude fat and gross energy were determined, according to AOAC [47] methods. The amino acid composition of diets are the respective estimated available proportions. Fatty acid methyl esters (FAME) of the macroalga and experimental diets were attained by one-step extraction and acidic transesterification, and analysed using a gas chromatograph with a flame ionization detector (HP7890A Hewlett-Packard, Avondale, PA) incorporated with a Supelcowax® 10 capillary column (30 m × 0.20 mm i.d., 0.20 μm film thickness; Supelco, Bellefonte, PA, USA), following the conditions described by Alfaia et al. [46]. The internal standard was the nonadecanoic acid (C19:0) methyl ester. Fatty acids were expressed as % of total fatty acids.

For the analysis of β-carotene and diterpenes (vitamin E homologs - tocopherols and tocotrienols), samples of *L. digitata* and diets (100 mg each) were weighed (750 mg) in duplicate and the above compounds were extracted as reported by Pestana et al. [30] and Prates et al. [30]. Samples were added with ascorbic acid followed by a saponification solution and were incubated and stirred in a water bath at 80 °C for 15 min. After saponification, n-hexane phases were separated by centrifugation (2500 rpm, 10 min), filtered, and then analysed in an HPLC system incorporated with a normal-phase silica column (Zorbax RX-Sil, 250 mm × 4.6 mm i.d., 5 μm particle size, Agilent Technologies Inc., Palo Alto, CA) and 2 detectors set on series, according to conditions previously described [46, 48]. The compounds were determined based on the external standard technique and using a standard curve of peak area versus concentration.

The analysis of pigments of *L. digitata* and experimental diets was performed according to Teimouri et al. [49] with minor modifications introduced by Pestana et al. [30]. Briefly, 0.5 g of samples were incubated overnight with 5 mL acetone and stirred in the dark. Afterwards, solutions subjected to centrifugation (at 4000 rpm for 5 min) and the absorbance at differential wavelengths (662 nm and 645 nm for chlorophylls a and b, and 470 nm for total carotenoids) was determined by UV-Vis spectrophotometry (Ultrospec 3100 pro, Amersham Biosciences, Little Chalfont, UK). The pigments content was determined using Hynstova et al. [50] equations.

The mineral profile of *L. digitata* and experimental diets, except for the bromine that was analysed according to Delgado et al. [51], was done exactly as Ribeiro et al. [52]. In brief, 0.3 g of samples were weighed in a digestion tube and added 3 mL of nitric acid (65%) and 10 mL of hydrochloric acid (37%). After, samples were incubated in a ventilated chamber during 16 h followed by the addition of 1 mL of hydrogen peroxide (30%). Afterwards, samples were heated using a digestion plate (DigiPREP MS, SCP Science, Quebec, Canada) 1 h to reach 95 °C and 1 h at 95 °C. Then, samples were left to cool and then diluted with distilled water for a final volume of 25 mL and filtered through filter papers (with 90 mm diameter) into sealed flasks. The solution was then analysed for the different elements by Inductively Coupled Plasma – Optical Emission Spectrometry (ICP-OES, iCAP 7200 duo Thermo Scientific, Waltham, MA, USA), using appropriate standards and calibration curves.

### Broilers performance, slaughtering and sampling

Birds were fed ad libitum, using a trough feeder, on a daily basis. Animals and feeders were weighed once a week to obtain ADFI, ADG and feed conversion ratio (FCR). At the end of the experimental period, one 35-day-old broiler per pen was slaughtered by electrical stunning and exsanguination. Carcasses were air-chilled and monitored with a probe thermometer until an internal temperature of 4 °C. For the analysis of pigments, diterpenes, fatty acids and minerals, liver samples were removed from the left side of carcasses, minced and stored at − 20 °C.

### Plasma biochemical markers

Plasma metabolites reflect lipid profile, glucose, urea, creatinine, total protein and liver function. To assess glucose, triacylglycerols (TAG), urea, creatinine, total cholesterol, HDL-cholesterol, LDL-cholesterol and total protein levels as well as the enzymatic activities of alanine aminotransferase (ALT, EC 2.6.1.2), aspartate aminotransferase (AST, EC 2.6.1.1), alkaline phosphatase (ALP, EC 3.1.3.1) and gamma-glutamyltransferase (GGT, EC 2.3.2.13) Modular Hitachi Analytical System diagnostic kits were applied (Roche Diagnostics, Mannheim, Germany). VLDL-cholesterol and total lipids were calculated by using Friedewald et al. [19] and Covaci et al. [18] formulas, respectively.

### Determination of total lipids and fatty acid composition in the liver

The extraction of total lipids from freeze-dried liver samples was performed, in duplicate, using dichloromethane: methanol (2:1, v/v) [53]. Lipids were determined gravimetrically. The fatty acids were converted to fatty acid methyl esters (FAME) by a combined alkaline and acidic transesterification [30]. Then, FAME were separated in a Supelcowax® 10 capillary column using gas chromatography (GC) with flame ionization detection (FID), and the running conditions were as previously described by Alfaia et al. [46]. Fatty acids were identified by comparison with a standard (FAME mix 37 compounds, Supelco Inc. Bellefonte, PA, USA), quantified using C19:0 methyl ester as an internal standard. Fatty acids were expressed as % of total fatty acids.

### Determination of total cholesterol, β-carotene, diterpenes and pigments in the liver

Total cholesterol, β-carotene and homologs of vitamin E were extracted, in duplicate, from liver samples (750 mg each), following the same procedure described for alga and diets, using direct saponification, single n-hexane extraction and HPLC analysis [30, 48].

The pigments (chlorophyll a, chlorophyll b and total carotenoids) in the liver samples were extracted as described for the macroalga and diets, but with minor modifications. Briefly, 2.5 g of liver were weighed and added 5 mL of acetone in the dark. This mixture was homogenized for 1 min and centrifuged (at 3000 rpm for 5 min at 4 °C). The supernatant was separated and immediately analysed, following the same procedure described by Coelho et al. [26]. The pigment contents were determined according to Hynstova et al. [50].

The mineral profile in the liver samples was determined as mentioned earlier for macroalga and diets.

### Statistical analysis

Data analysis was performed by ANOVA from Generalized Linear Mixed (GLM) model of Statistical Analysis System (SAS) program (SAS Institute Inc., Cary, NC) and by the adjusted Tukey-Kramer method (PDIFF option) for multiple comparisons of least squares means [54]. In addition, the PROC POWER model of SAS was used for evaluation of statistical power. The experimental unit was either the cage (for feed intake and feed conversion ratio) or the bird (for body weight, body weight gain, plasma metabolites and overall hepatic parameters). The treatment was considered a fixed factor in the model. All statistical tests were considered significant at a probability level of 5%.

A principal component analysis (PCA) was completed with hepatic fatty acids, antioxidants and pigments, and minerals from broilers. To do so, the Statistica program (version 8.0; TIBCO software, Palo Alto, CA, USA) was applied to a data set of 40 samples and 45 variables to reduce the dimensionality of the data set and to describe the variability of data into two dimensions. After data normalization, the principal components were accepted as significant if they contributed more than 5% for the total variance.

## Data Availability

All data produced in this study are included in the published version. Datasets are accessible from the corresponding author on request.

## Abbreviations

ADFI: Average daily feed intake
ADG: Average daily gain
ALT: Alanine aminotransferase
ALP: Alkaline phosphatase
AST: Aspartate aminotransferase
CAZymes: Carbohydrate-active enzymes
FAME: Fatty acid methyl esters
FCR: Feed conversion ratio
GGT: Gamma-glutamyltransferase
HDL: High-density lipoprotein cholesterol
LDL: Low-density lipoprotein cholesterol
MUFA: Monounsaturated fatty acids
PC: Principal components
PCA: Principal component analysis
PUFA: Polyunsaturated fatty acids
SFA: Saturated fatty acids
TAG: Triacylglycerols
VLDL: Very-low-density lipoprotein cholesterol
